# Characterization of plasmid-mediated quinolone resistance genes and extended-spectrum beta-lactamases in non-typhoidal *Salmonella enterica* isolated from broiler chickens

**DOI:** 10.14202/vetworld.2022.1515-1522

**Published:** 2022-06-18

**Authors:** Esraa Razzaq Hassan, Abdullah O. Alhatami, Husam Muhsen Abdulwahab, Bradly S. Schneider

**Affiliations:** 1 Department of Microbiology, Faculty of Veterinary Medicine, University of Kufa, Kufa, Iraq; 2 Department of Public Health, Faculty of Veterinary Medicine, University of Kufa, Kufa, Iraq; 3 Department of Pathology, Faculty of Veterinary Medicine, University of Kufa, Kufa, Iraq; 4 Etiologic, San Francisco, California, United States

**Keywords:** antimicrobial resistance, extended-spectrum β-lactamases, food-borne pathogen, multidrug-resistant, poultry, *qnr*, *Salmonella*.

## Abstract

**Background and Aim:**

Antibiotic-resistant *Salmonella* is a public health concern. Fluoroquinolones and extended-spectrum beta-lactams are widely used for the treatment of *Salmonella* infections. This study focused on the detection of plasmid-mediated quinolone resistance (PMQR) and extended-spectrum beta-lactamase (ESBL) genes among multidrug-resistant (MDR) *Salmonella enterica* isolated from broilers.

**Materials and Methods:**

A total of 40 non-typhoidal *S. enterica* isolates were collected from 28 broiler chicken farms in four Iraqi Governorates. These isolates were examined for their susceptibility to 10 antimicrobial agents by disk-diffusion method followed by polymerase chain reaction assay for the detection of PMQR determinants and ESBLs genes.

**Results:**

*Salmonella* strains revealed high levels of resistance to the following antibiotics: Nalidixic acid 100%, levofloxacin (LEV) 97.5%, amoxicillin-clavulanic acid 95.0%, tetracycline 92.5%, and nitrofurantoin 80.0%. Otherwise, all isolates were susceptible to cefotaxime and ceftriaxone. All isolates were MDR, with 15 different profiles observed. Among 38 amoxicillin/clavulanic acid-resistant *Salmonella* isolates, 20 (52.6%) had the *bla*TEM gene, while *bla*SHV, *bla*CTX-M, and *bla*OXA genes were not detected. Only 5 (12.8%) out of 39 LEV-resistant isolates were positive for *qnrB*, three of which had *bla*TEM. No *qnrC* or *qnrD, qnrS*, *aac(6`)-Ib-cr*, *qunA*, and *oqxAB* genes were found in any of the tested isolates.

**Conclusion:**

This study demonstrates that broiler chickens may be considered a potential source for spreading MDR non-typhoidal *Salmonella* and ESBL traits in poultry production. Therefore, it is important to continuously monitor ESBL and PMQR genes to avoid the spread of resistant strains in the food chain and impact public health.

## Introduction

Non-typhoidal *Salmonella* (NTS) is one of the main causes of diarrhea in humans and is transmitted primarily by contaminated food of animal origin. In developing countries, poultry and domestic animals are considered the main reservoirs of NTS, which can also impact human health [1]. Food-borne infections caused by *Salmonella* spp. are increasing in many countries [2]. During the past 40 years, antimicrobial drug-resistant strains have been reported within different serotypes of *Salmonella enterica* [3]. The emergence and spread of multidrug-resistant (MDR) *Salmonella* in the food chain represents a great threat to human health [4]. Moreover, there is an increase in the prevalence of MDR *Salmonella* serotypes that are recovered from humans and animals, particularly resistant strains for clinically important antimicrobials [5, 6]. Several zoonotic serotypes such as *Salmonella* Enteritidis, *Salmonella* Typhimurium, *Salmonella* Virchow, and *Salmonella* Hadar have developed different drug resistance patterns. Of particular significance, the phage-type DT104 of *S*. Typhimurium with a specific pattern of resistance to ampicillin, chloramphenicol (C), streptomycin, sulfonamides, and tetracycline (TE)was clonally disseminated throughout the world starting in the 1990s [7]. There are several factors that contribute to the development of antibiotic resistance in *Salmonella*, including chromosomal mutations, which usually lead to resistance to one drug, such as nalidixic acid (NA) resistance, the overuse and misuse of antibiotics may enhance the selection of resistant mutants and spread of such strains in animals or humans, and the transmissible genetic materials (R plasmids, transposons, integrons, and *Salmonella* genomic islands). These factors and elements are potentially correlated with the emergence and spread of antibiotic resistance genes in *S. enterica* [8, 9]. In addition, R plasmids can carry virulence genes like the toxin production gene, which confers increased virulence. Thus, the use of antibiotics may select for bacteria carrying plasmids that confer multiple drug resistance and increased pathogenicity [10].

Extended-spectrum beta-lactamases (ESBLs)-producing *Salmonella* isolates have been commonly isolated from food animals in numerous countries [11, 12]. ESBLs belong to Group 2 of the functional classification and Classes A and D of the molecular classification. Previously, TEM-(the abbreviation of the name belongs to a patient called Temoniera), and SHV-type (means sulfhydryl variable) enzymes were prevalent in the Enterobacteriaceae family members [13]. However, currently, other forms of ESBLs enzymes such as CTX-M (cefotaxime [CTX] resistance gene and -M from Munich) and OXA (oxacillin hydrolysis enzyme) are the most prevalent types of ESBLs in Gram-negative bacteria [14–16]. ESBL enzymes are usually encoded by genes carried on conjugative plasmids and harbored by transmissible genetic elements such as insertion sequences and transposons that speed up their dissemination in bacterial community [17]. Quinolones and fluoroquinolones are broad-spectrum antimicrobial agents extensively used in poultry disease treatment. This widespread use has been associated with a worldwide increase in levels of resistance to such agents, especially in Gram-negative bacteria species in the past decade [18, 19]. Globally, many serotypes of *S. enterica* have developed resistance to NA and reduced susceptibility to fluoroquinolones due to chromosomally mediated mutations in the quinolone resistance-determining regions of the DNA gyrase and topoisomerase IV genes that lead to target modification [20]. Recently, quinolone resistance was found in *Salmonella* to be mediated by the acquisition of plasmid-encoded genes (plasmid-mediated quinolone resistance [PMQR]), including *qnr* genes, efflux pump mechanisms (*qepA* and *oqxAB*), and fluoroquinolone-modifying enzyme (aminoglycoside acetyltransferase) encoded by the *aac (6’) Ib-cr* gene [21–23]. Equally important, several researchers have detected *qnr* genes and ESBLs in the same bacterial isolates [22–24], this coexistence of resistance determinants may select for quinolone resistance and increase the prevalence of ESBL genes in the bacterial population [25–27]. In Iraq, the molecular characterization of PMQR and ESBL genes in *Salmonella* from broiler chickens has not been conducted. Therefore, the detection of such resistance genes has veterinary and medical importance to guide the implementation of surveillance and control programs, locally and on a national scale.

In the present study, MDR strains of NTS isolated from broiler chickens in Middle Euphrates region of Iraq were screened for PMQR (*qnrA*, *qepA*, *oqxAB*, *qnrC*, *qnrB*, *qnrS*, *qunD*, and *aac(6`)-Ib-cr*) and ESBL (*bla*TEM, *bla*SHV, *bla*CTX-M, and *bla*OXA) genes.

## Material and Methods

### Ethical approval

Fecal cloacal swabs were collected from broiler chickens as part of normal surveillance. The farm owners gave oral permission for their farms to be included in this study. No interventions were needed in this study, so there is no need for ethical approval.

### Study period and location

This study was conducted from October 2017 to March 2018 in four Middle Euphrates Governorates (Al-Najaf, Al-Muthanna, Al-Qadisiyyah, and Babylon).

### Bacterial isolates

This study only included chickens from broiler farms. A total of 40 NTS isolates were recovered from 37 flocks (five samples for each flock, 185 samples total) that belong to 28 broiler farms. Briefly, all NTS isolates were incubated by enrichment method and subcultured onto a chromogenic medium (CHROMagar Company, Paris, France). Next, suspected colonies were identified by Gram staining; biochemical identification using Simmons citrate, triple sugar iron, urease, and lysine iron agar tests [28]; and *invA* gene amplification by polymerase chain reaction (PCR), as previously described [29, 30]. Isolates were then stored in Luria-Bertani (LB) broth (Oxoid, UK) with 15% glycerol at −20°C. Frozen isolates were thawed and streaked on CHROMagar *Salmonella* agar (CHROMagar Company).

### Antimicrobial susceptibility testing

Measurement of the antibiotic susceptibility of NTS isolates was performed by the standard disk-diffusion method in Mueller-Hinton agar (Himedia, India) in accordance with guidelines recommended by Clinical and Laboratory Standards [26]. Antimicrobials tested were ampicillin (AMP, 30 μg), amoxicillin/clavulanic (AMC, 20/10 μg), CTX (30 μg), ceftriaxone (CRO, 30 μg), C (30 μg), TE (30 μg), levofloxacin (LEV, 5 μg), NA (30 μg), trimethoprim (TMP, 5 μg), and nitrofurantoin (F, 300 μg). All the disks were purchased from Bioanalyse Company, Turkey. The inhibition zones were recorded, and the results were interpreted according to the criteria of the Clinical and Laboratory Standards Institute [31]. *Escherichia coli* ATCC 25922 was used for quality control. MDR *Salmonella* was defined as resistance to three or more antibiotic classes [32].

### DNA extraction and purification

The strains were streaked on LB agar and incubated overnight at 37°C. Genomic DNA was extracted using Genomic DNA Mini Kit (Blood/Cultured Cell, Geneaid, USA), according to the manufacturer’s instructions.

### PCR screening for the PMQR genes

The LEV-resistant isolates were screened for PMQR coding genes by multiplex PCR for *qnrA, qepA*, *oqxAB*, and *qnrC* and monoplex PCR for *qnrB, qnrS*, *qnrD*, and *aac(6`)-Ib-cr*, using specific primers (Table-1) and conditions described by the previous studies [33–37]. The PCR was performed with an Agilent Sure Cycler 8800, thermocycler (Agilent Technologies, USA). The reaction was carried out in a volume of 25 μL containing 1 μL of forward and reverse primer, 5 μL DNA, 5 μL molecular grade water, and 12.5 μL of BlasTaq™ 2× PCR MasterMix (Applied Biological Materials, Canada). Amplification of the *invA* gene (housekeeping gene) was used as a reaction positive control for all PCR reactions. The primer sequence and amplification protocol were used as per mentioned previously [29].

**Table 1:** Primer sequences for the eight plasmid-mediated resistance gene determinants.

Primer name	Sequence (5’ to 3’)	Product size/bp	Reference
*qnrA*			
F	CAGCAAGAGGATTTCTCACG	630	[34]
R	AATCCGGCAGCACTATTACTC		
*qnrD*			
F	CGAGATCAATTTACGGGGAATA	582 bp	[33]
R	AACAAGCTGAAGCGCCTG		
*qnrB*			
F	GATCGTGAAAGCCAGAAAGG	469 bp	[35]
R	ACGATGCCTGGTAGTTGTCC		
*qnrS*			
F	ACGACATTCGTCAACTGCAA	417 bp	
R	TAAATTGGCACCCTGTAGGC		
*oqxAB*			
F	CCGCACCGATAAATTAGTCCGGCG	313 bp	[34]
R	AGGTTTTGATAGTGGA		
*aac (6’)-Ib-cr*			
F	TATGAGTGGCTAAATCGAT	395 bp	[36]
R	CCCGCTTTCTCGTAGCA		
*qepA*			
F	GCAGGTCCAGCAGCGGGTAG	218 bp	[37]
R	CTTCCTGCCCGAGTATCGTG		
*qnrC*			
F	GCAGAATTCAGGGGTGTGAT	118 bp	[34]
R	AACTGCTCCAAAAGCTGCTC		

### PCR screening for beta-lactamase gene families

All AMC-resistant *S. enterica* isolates were analyzed by multiplex and singleplex PCR with specific primers (Table-2) for the detection of ESBL-encoding genes, namely, *bla*TEM, *bla*SHV, *bla*CTX-M, and *bla*OXA, respectively. The reaction mixture was assembled according to methods described previously [38, 39].

**Table 2:** Sequence of the oligonucleotide primers used for the detection of extended-spectrum beta-lactamase genes.

Primer name	Sequence (5’ to 3’)	Product size (bp)	Reference
*bla* SHV			
F	CGCCTGTGTATTATCTCCCT	293 bp	[39]
R	CGAGTAGTCCACCAGATCCT		
*bla* TEM			
F	TTTCGTGTCGCCCTTATTCC	403 bp	
R	ATCGTTGTCAGAAGTAAGTTGG		
*bla* CTX-M			
F	CGCTGTTGTTAGGAAGTGTG	569 bp	
R	GGCTGGGTGAAGTAAGTGAC		
*bla* OXA			
F	ACCAGATTCAACTTTCAA	598 bp	[38]
R	TCTTGGCTTTTATGCTTG		

### Statistical analysis

The Chi-square test was used to determine any significant differences in resistance. Differences were considered significant at p < 0.05. Results were formulated as tables and figures where appropriate.

## Results

*S. enterica* isolates showed high levels of resistance to NA (100%), LEV (97.5%), amoxicillin-clavulanic acid (95%), TE (92.5%), and F (80%), as presented in Table-3. The statistical analysis demonstrated that these resistance rates were significantly high (p < 0.01). A moderate resistance rate was observed to C (60%) (p < 0.05). The lowest resistance rate was observed to ampicillin (45%) and TMP/sulfamethoxazole (12.5%). All isolates were MDR, with a total of 15 different patterns observed (Table-4). Thirty-four isolates were simultaneously resistant to TE, NA, AMC, and LEV. On the other hand, all isolates were susceptible to CTX and CRO.

**Table 3:** Antibiogram of NTS strains collected from broiler farms by disk diffusion method.

Antimicrobial agent	Resistance (%)	Susceptibility (%)
Ampicillin	18 (45)	22 (55)*
Amoxicillin/clavulanic	38 (95)**	2 (5)
Cefotaxime	0	40 (100)**
Ceftriaxone	0	40 (100)**
Chloramphenicol	24 (60)*	16 (40)
Tetracycline	37 (92.5)**	3 (7.5)
Levofloxacin	39 (97.5)**	1 (2.5)
Nalidixic acid	40 (100)**	0
Trimethoprim	20 (50)*	20 (50)
Nitrofurantoin	32 (80)**	8 (20)

* p < 0.05,

** p < 0.01

**Table 4:** Antibiotic resistance patterns of MDR *Salmonella* isolates of broilers.

Resistance pattern	No. of isolates	Resistance gene pattern
AMC/F/LEV/NA/TE/TMP	3	-
	1	*bla* TEM
AM/AMC/C/F/LEV/NA/TE/TMP	6	*bla* TEM
AMC/C/F/LEV/NA/TE	2	-
	1	*bla* TEM
AMC/C/F/LEV/NA/TE/TMP	4	-
	1	*qnrB*
AM/AMC/C/F/LEV/NA/TE	5	*bla* TEM
AMC/LEV/NA/TE/TMP	1	-
AMC/F/LEV/NA/TMP	1	-
AMC/F/NA/TE	1	*-*
AM/AMC/C/LEV//NA/TE	3	*bla* TEM
	1	*bla* TEM, *qnrB*
AM/AMC/F/LEV/NA/TE	1	*bla* TEM
F/LEV/NA/TE	2	*-*
AMC/F/LEV/NA/TE	1	*qnrB*
	2	-
AM/AMC/LEV/NA/TE/TMP	1	*bla* TEM, *qnrB*
	1	*bla* TEM
AMC/F/NA	1	-
AMC/C/F/LEV/NA	1	*-*

*AM=Ampicillin, AMC=Amoxicillin/clavulanic, C=Chloramphenicol, F=Nitrofurantoin, LEV=Levofloxacin, NA=Nalidixic acid, TE=Tetracycline, TMP=Trimethoprim

**Figure-1:**
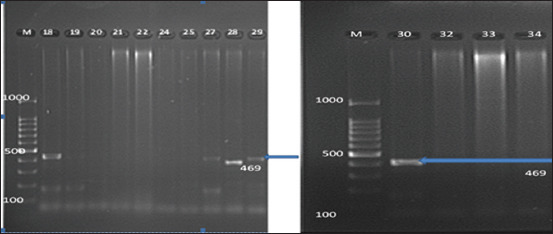
Amplification of *qnrB* gene by a polymerase chain reaction from *Salmonella* isolates. Ethidium bromide-stained agarose gel (75 V at 1 h). Lane M, DNA molecular size marker (100 bp ladder), lanes 18, 27, 28, 29, and 30 show positive results *qnrB* gene (469 bp).

Among 39 LEV-resistant isolates screened for PMQR genes, 5 (12.8%) isolates were positive for *qnrB* (Figure-1); no *qnrC*, *qnrD, qnrS*, *aac(6`)-Ib-cr*, *qunA*, or *oqxAB* genes were found in any of the tested isolates. Moreover, three of *qnrB*-positive isolates amplified *bla*TEM.

**Figure-2:**
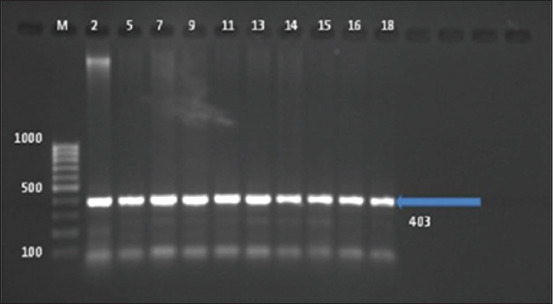
Amplification of *bla*SHV, *bla*TEM, and *bla*CTX-M genes by a multiplex polymerase chain reaction from *Salmonella* isolates. Ethidium bromide-stained agarose gel (75 V at 1 h). Lane M, DNA molecular size marker (100 bp ladder); lanes 2, 5, 7, 9, 11, 13, 14, 15, 16, and 18 positive with *bla*TEM gene (403 bp).

Regarding ESBLs gene PCR amplification, 52.6% (20/38) of resistant isolates to AMC acid were amplified *bla*TEM gene products (Figure-2). However, no amplified products were detected for *bla*SHV, *bla*CTX-M, and *bla*OXA, in any isolate.

## Discussion

Antibiotic resistance in *Salmonella* and the emergence of MDR isolates are becoming a great public concern worldwide [40]. In the present study, higher resistance rates were observed for NA and TE, which agrees with reports among broiler chicken isolates from many parts of the world [41], including Zishiri *et al*. [42], who reported similar levels of TE resistance (93%). These results could be due to misuse and wide use of TE in poultry for treatment, prophylaxis, and as a growth promoter in Iraq. NA is not commonly used in poultry, but the resistance observed could be related to the wide use of enrofloxacin and other quinolone derivatives [43]. In contrast, other researchers have found lower levels of resistance to TE (11%) and NA (0%) among *Salmonella* recovered from chicken droppings in Nairobi, Kenya [44].

Fluoroquinolones and F are not only used in veterinary practice but also in human medicine to treat various bacterial infections. Unfortunately, a high prevalence rate of resistance to LEV and F (97.5% and 80%, respectively) was detected in the present study. These results are similar to that reported in other studies from Asia, Europe, South America, and North America [45–48]. In the present study, the increased resistance to F was expected due to its large-scale usage in poultry production at both therapeutic and subtherapeutic doses (due to incorporation with feed or as growth promoters) [49]. There are several genetic mechanisms that confer F resistance, including chromosomally mediated mutations in nfsA, nfsB, and ribE proteins, as well as plasmid-mediated multiple drug efflux pumps that encode by *oqxAB* genes [50]. Therefore, F resistance may represent an indicator of extensive antibiotic resistance in enteric bacteria [51].

Despite the infrequent use of C in poultry farming in Iraq, about 60% of the present isolates showed resistance to this antibiotic. These results are in alignment with observations of earlier studies [52]. This could be due to the fact that previous continuous exposure to this antibiotic may result in the development of resistant strains, which may persist for years in the ecosystem even after discontinuation [53].

The current observation of high levels of amoxicillin-clavulanic acid resistance among *Salmonella* isolates does not align with expectations. Further confirmatory work will be required to measure the minimal inhibitory concentration of amoxicillin-clavulanic acid to find out if these isolates have moderate susceptibility [54].

Fortunately, the present findings showed that all isolates were susceptible to CTX and CRO, which may be because these antimicrobials are less commonly used for therapeutic purposes in veterinary medicine or as a growth promoter in conventional animal fattening.

The development of MDR zoonotic bacteria represents a multifaceted risk, as they pose a threat to animal productivity and food security, as well as public health through their transmission through the food chain to humans. In addition, these genes may horizontally transfer resistance to other pathogens [55].

All the present isolates were resistant to three or more different classes of antibiotics. Several studies have found an increased prevalence of MDR among *Salmonella* recovered from poultry [56, 57]. Notably, in China, a high prevalence rate (81.1%) of MDR *Salmonella* isolated from chickens was reported [58]. A Slovenian study found that 88.5% of *Salmonella* Infantis isolates were MDR [59]. Moreover, numerous studies have found an increased prevalence of MDR isolates of NTS in many parts of the world [60]. Equally important, the present study characterized 15 patterns of MDR. This suggests that there is high antibiotic pressure in the area of this study that led to the development of novel profiles of resistance beyond the classical pattern of MDR usually seen in *S*. Typhimurium. The increasing prevalence of MDR *S. enterica* could thus lead to the emergence of superbug salmonellae [3].

The present study focused on the PMQR genes. The *qnrB* gene was observed in low prevalence (12.8%) among LEV-resistant strains. This suggests that other mechanisms could be implicated in fluoroquinolones resistance, such as chromosomal mutations that target DNA gyrase and topoisomerase IV [61]. These findings agree with a previous study conducted by Yang *et al*. [62], while differing from the study conducted by Ata *et al*. [63].

Furthermore, the current research characterized three isolates that amplified *bla*TEM and *qnrB*. This is an alarming indicator of the prevalence of PMQR genes in NTS strains isolated from poultry in Iraq. The World Health Organization (WHO) published a priority list of antibiotic-resistant bacteria to help in prioritizing the research and development of new effective antibacterial therapy. *Salmonella* spp., particularly fluoroquinolone-resistant strains, were one of the highest priority pathogens. The WHO encourages field surveys on livestock and data sharing between human and animal health sectors. These efforts will help to reduce the risk of antibiotic resistance and increase the effectiveness of One Health approaches in reducing the spread of antimicrobial resistance [64].

There are several mechanisms for beta-lactam resistance, mainly the production of beta-lactamases that hydrolyze the beta-lactam ring and inhibit the activity of such antibacterial agents [65]. Many ESBL genes have been detected in *Enterobacteriaceae* isolated from poultry [66, 67]. We detected only *bla*TEM in most AMC acid-resistant isolates. This was similar to the previous studies that detected TEM only [68, 69] or reported predominance of TEM [44, 66]. On the contrary, other researchers have reported the predominance of beta-lactamase genes such as *OXA*, *CMY*-2, and *CTX*-M [70–72].

It is difficult to explain the variation in the prevalence of beta-lactamase genes in *Salmonella* isolated from poultry throughout the world. A common explanation is that it is related to the injudicious use of antibiotics, which may create a selective pressure that selects for the acquisition of specific beta-lactamase genes. Some of the AMC acid-resistant isolates in our study did not have any of the tested beta-lactamases genes. This could be due to other beta-lactamases genes, such as *bla*CMY, *bla*PSE, or other resistance mechanisms.

## Conclusion

This study found that all isolates detected were MDR and most of the isolates displayed multiple resistance to nalidixic, LEV, amoxicillin-clavulanic acid, and TE. In addition, we observed the predominance of *bla*TEM among other ESBL-encoding genes in this study, which have a potential risk to human health. The PMQR and ESBLs in NTS should be continuously monitored to avoid the spread of such resistant strains in the food chain, which may impact public health. The prevalence of MDR *Salmonella* should be controlled through the implementation of educational programs covering the indications for prescribing antibiotics and the optimal duration of use for the treatment of bacterial infections in poultry production, as well as antibiotic stewardship programs and training on the use of prebiotics, probiotics, acidifiers, and phage therapy to control colonization of *Salmonella* and treatment of infections as an alternative to antibiotics.

## Authors’ Contributions

AOA: Designed the study and critically revised the manuscript. ERH: Collected samples and performed bacteriological isolation and identification, genomic DNA extraction, and PCR testing. HMA: Collected samples, performed antibiotic susceptibility testing, and interpreted the results. BSS: Drafted, revised, and finalized the manuscript for submission. All authors have read and approved the final manuscript.
